# Biofilm Formation on Excavation Damaged Zone Fractures in Deep Neogene Sedimentary Rock

**DOI:** 10.1007/s00248-024-02451-7

**Published:** 2024-10-22

**Authors:** Akinari Hirota, Mariko Kouduka, Akari Fukuda, Kazuya Miyakawa, Keisuke Sakuma, Yusuke Ozaki, Eiichi Ishii, Yohey Suzuki

**Affiliations:** 1 Regulatory Standard and Research Department, Secretariat of Nuclear Regulation Authority (S/NRA/R), 1-9-9, Roppongi, Minato-Ku, Tokyo, 106-8450 Japan; 2 Department of Earth and Planetary Science, The University of Tokyo, 7-3-1 Hongo, Bunkyo-Ku, Tokyo, Japan; 3 Horonobe Underground Research Center, Japan Atomic Energy Agency, 432-2 Hokushin, Horonobe-Cho, Hokkaido, 098-3224 Japan; 4 Nuclear Safety Research Center, Japan Atomic Energy Agency, 2-4 Shirakata, Tokai-Mura, Naka-Gun, Ibaraki, 319-1195 Japan

**Keywords:** Biofilm Formation, Excavation Damaged Zone, Radioactive Waste Disposal, Aerobic Methanotrophs, Dark O_2_

## Abstract

Deep underground galleries are used to access the deep biosphere in addition to mining and other engineering applications, such as geological disposal of radioactive waste. Fracture networks developed in the excavation damaged zone (EDZ) are concerned with accelerating mass transport, where microbial colonization might be possible due to the availability of space and nutrients. In this study, microbial biofilms at EDZ fractures were investigated by drilling from a 350-m-deep gallery and subsequent borehole logging at the Horonobe Underground Research Laboratory (URL). By using microscopic and spectroscopic techniques, the dense colonization of microbial cells was demonstrated at the surfaces of the EDZ fractures with high hydraulic conductivity. 16S rRNA gene sequence analysis revealed the dominance of gammaproteobacterial lineages, the cultivated members of which are aerobic methanotrophs. The near-complete genomes from Horonobe groundwater, affiliated with the methanotrophic lineages, were fully equipped with genes involved in aerobic methanotrophy. Although the mediation of aerobic methanotrophy remains to be demonstrated, microbial O_2_ production was supported by the presence of genes in the near-complete genomes, such as catalase and superoxide dismutase that produce O_2_ from reactive oxygen species and a nitric oxide reductase gene with the substitutions of amino acids in motifs. It is concluded that the EDZ fractures provide energetically favorable subsurface habitats for microorganisms.

**Supplementary Information:**

The online version contains supplementary material available at 10.1007/s00248-024-02451-7.

## Introduction

For sampling microbes from the deep subsurface, drilling from the ground surface is problematic due to the disturbance induced by drilling fluid contamination. To reduce the disturbance level after drilling, groundwater needs to be pumped out from boreholes for the removal of drilling fluid contamination. Our previous study demonstrated that drilling fluid contamination significantly changed the microbial communities in a borehole drilled from the ground surface even after pumping a large amount of groundwater [1]. In contrast, boreholes drilled from underground facilities are suitable for geochemical and microbiological investigations, because it is easy to remove drilling fluid contamination by flushing a large amount of groundwater without using a pump system [2, 3]. Recent metagenomic analysis of high-quality groundwater samples from boreholes drilled from underground facilities has expanded our understanding of taxonomic and metabolic profiles of subsurface microbes [4–7]. Therefore, underground facilities are regarded as windows to the deep biosphere.

The subsurface construction of large open spaces for human activities is known to change the hydromechanical properties of the surrounding geological formations due to stress redistribution. An “excavation damaged zone (EDZ)” is formed in geological formations with variable extents of permeability and consolidation at several tens of centimeters and at most, less than 3 m from underground openings [8, 9]. The EDZ is typically associated with hydromechanical and geochemical modifications, such as an increase in hydraulic conductivity by one or more orders of magnitude [9]. Numerous studies have been conducted for the thermo-hydro-mechanical-chemical (THMC) behavior of EDZs in various rock types (e.g., [9–20]). However, the microbial behavior at an EDZ is poorly understood. Given that the growth of subsurface microbes is stimulated by the supply of substrates through fractures, it might be possible to perform metagenomic analysis of the microbial communities in rock core samples.

Generally, sorption processes delay radionuclide transport, and redox conditions are a key geochemical factor influencing sorption capabilities, particularly for redox-sensitive radionuclides (e.g., radioisotopes of U and Np) (e.g., [8, 21, 22]). Consequently, changes in redox conditions are a concern, as they can affect radionuclide transport and ultimately impact the safety of a repository. If microbes thrive in EDZ fractures, investigations of the bioavailability of electron donors and acceptors may provide useful information on the redox conditions in rocks around the underground facilities during an operation. Additionally, the metabolic activities of microbes could be used to predict shifts in redox conditions after the closure.

Our recent studies of the microbiomes in the groundwater from the boreholes drilled from two Japanese underground research facilities for radioactive waste repositories revealed the dominance of anaerobic methane (CH_4_)-oxidizing archaea that reduce Fe(III) and sulfate [5, 6]. These results highlight the importance of methane in the deep geological formations. Since the groundwater samples were collected from boreholes at least 10 m apart from the underground facilities, microbes in the rocks near the underground facilities might similarly depend on CH_4_ for the energy source. To test this hypothesis, we conducted a 2-m-long coring and borehole logging at a 350-m-deep gallery at one of the two Japanese underground facilities called the Horonobe Underground Research Laboratory (URL). As dense microbial colonization was evident at EDZ fractures, microbial biomasses separated from the EDZ fractures were subjected to 16S rRNA gene amplicon analysis and subsequent genomic analysis to obtain taxonomic and metabolic profiles.

## Materials and Methods

### Horonobe URL

**Fig. 1:**
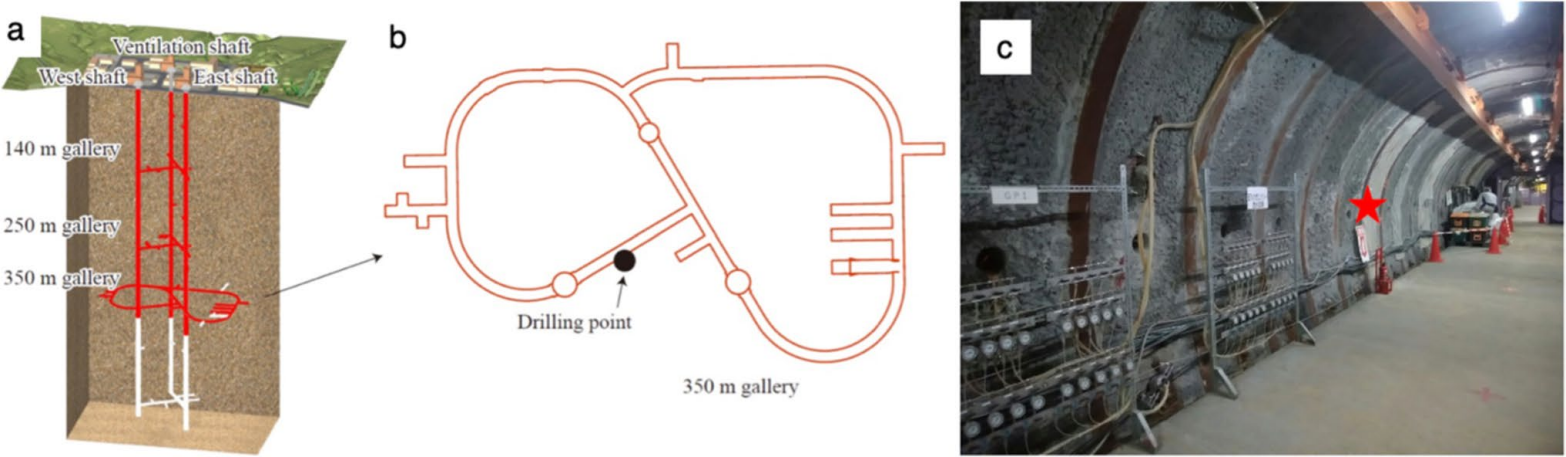
A schematic illustration showing the Horonobe Underground Research Laboratory (URL) at the time of sample collection (a). The horizontal layout of 350-m-deep galleries with drilling point at the time of sample collection (b). A picture of a 350-m-deep gallery used for drilling (c). The drilling point is indicated by a red star

Horonobe URL is located in Horonobe town, Hokkaido, Japan, to enhance the reliability of technologies for the radioactive waste disposal in sedimentary rocks (Fig. 1). Horonobe URL consists of a ventilation shaft, east access shaft, and west access shaft with horizontal galleries excavated at depths of 140, 250, and 350 mbgl (mbgl: meters below ground level). The construction of the 350-m gallery was completed in June 2014. The sedimentary rocks around the URL consist of the Wakkanai Formation (Neogene siliceous mudstones) overlain by the Koetoi Formation (Neogene to Quaternary diatomaceous mudstones) with a transition zone (~ 200 − 450 mbgl) [24]. The origin of < 350-m-deep groundwater is fossil seawater mostly diluted by meteoric water [25–27]. As gallery walls were cemented at their surfaces, water seepage into the gallery walls is very limited at the three gallery depths.

Groundwater samples from the boreholes drilled from the 350-m gallery are highly reducing with Eh values below − 150 mV. In addition, the groundwater samples contained saturated levels of CH_4_, (up to 20 mmol kg^−1^). The concentrations of SO_4_^2−^ and NO_3_^−^ are low, at approximately 1 µmol kg^−1^ and 20 µmol kg^−1^ or below, respectively [28–30]. The depletion of these potential electron acceptors indicates that the groundwater water was anaerobic. The concentrations of the potential electron donors, NH_4_^+^ and HS^−^, are approximately several mmol kg^−1^ and below 1 µmol kg^−1^, respectively [30].

### Drilling and Borehole Logging

A 2-m-long borehole was horizontally drilled using an electro-hydraulic core drill at a 350-m gallery in January and February 2022 (Fig. 1). As drilling fluid, we used groundwater at a depth of approximately 50 m in Horonobe town [31]. A double-core barrel with a diameter of 86 mm was used for core sampling, and the core diameter was 65 mm. The collected cores were split in half with a rock cutter without using any fluid. The half-split cores were visually observed and vacuum-packed together with an oxygen absorber and then stored at − 30 °C. Borehole televiewer (BTV) observations were performed to measure the strikes and dips of fractures. A single-hole hydraulic test based on constant pressure step water injection was performed in six sections. For the low permeable rock, the result of a pulse test was used to estimate the hydraulic conductivity. Rock core samples from ~ 0.3 to ~ 0.5 m along the borehole (mabh) were frozen on-site for microbiological characterizations.

### Visualization of Microbial Cells from Rock Fractures

Drill core samples with fractures stored at − 30 °C were cut using a precision diamond band saw (DWS 3500P, Meiwafosis Co., Ltd., Japan) without using any fluid. Fracture and cut surfaces were stained with a fluorescence DNA dye called SYBR Green I (Takara Bio Inc., Japan) for microbial visualization. After washing with ultrapure water, the surfaces were observed using a fluorescence stereoscopic microscope (Leica Microsystems M205FA, German) with a color camera (Leica DFC310 FX, German) and the imaging software LAS V3.1. Materials attached to the surfaces were removed using flame-sterilized spatulas and attached to gelatin-coated slide glass. After staining with SYBR Green I, the materials covered with an anti-fading agent Vectashield (Vector Laboratories, USA) were observed using a fluorescence microscope (Olympus BX51, Olympus, Japan) with a CCD camera (Olympus DP71, Olympus, Japan) and the image processing software Lumina Vision (Mitani Shoji Co., Ltd.).

### Optical-Photothermal Infrared (O-PTIR) Spectroscopic Characterizations

O-PTIR spectra at a submicron resolution were obtained using a mIRage infrared microscope (Photothermal Spectroscopy Corp., Santa Barbara, USA) in refection mode (Cassegrain 40 objective (0.78 NA)) with a continuous wave (CW) 532 nm laser as the probe beam. To obtain O-PTIR spectra over the mid-IR ranges, a pump beam consisting of a tunable QCL device (800–1790 cm^−1^; 2 cm^−1^ spectral resolution and 10 scans per spectrum) was used. O-PTIR spectra from co-cultured cells of Nanoarchaeota strain MJ1 and *Metallosphaera* sp. strain MJ1HA (JCM33617) and cultured cells of *Shewanella oneidensis* (ATCC 700550) were obtained on disks made of CaF_2_. The average pf five O-PTIR spectra was calculated, and 4% IR laser power and 0.4% probe laser power were used to minimize sample damage.

### DNA Sequencing and Phylogenomic Analysis

Genomic DNA was extracted from materials obtained by scraping off the surfaces of EDZ fractures in the drill core samples with forceps. As the amount of the materials was limited, DNA extraction was performed from one sample per each EDZ fracture. Although the groundwater from approximately 50 m was used as drilling fluid, the groundwater was not sampled to assess microbial contamination. Instead, we scraped off the exterior surfaces of the drill core samples created by drilling, because that was where the microbial cells in the groundwater were attached. Then, we sampled microbes in the rock interior as a background by scraping off the surface created by cutting with a diamond band saw was also scraped off and subjecting the scraped material to DNA extraction. Approximately 0.1 g of the scraped material was incubated in an alkaline solution consisting of 0.5 M NaOH and TE buffer at 65 °C for 30 min (Nippon Gene Co., Japan) [32]. The supernatant was neutralized with 1 M Tris–HCl (pH 6.5; Nippon Gene Co., Japan) after centrifugation at 5000 × *g* for 30 s. After neutralization and ethanol precipitation with Ethachinmate (Nippon Gene Co., Japan), the precipitate was dissolved in 50 µl of TE buffer. The DNA extractants were stored in a TE buffer (pH 7.0–7.5) at − 4 °C or − 20 °C for longer storage. DNA extraction was performed without the addition of a subsample for negative control.

Using the primers Uni530F and Uni907R [33], 16S rRNA gene sequences were amplified by PCR using Ex Taq polymerase (Takara Bio, Inc., Shiga, Japan). In a reaction mixture containing 0.1 µM oligonucleotide primer and ca. 0.1 ng/µL DNA template, PCR was performed with 35 cycles of denaturation at 96 °C for 20 s, annealing at 56 °C for 45 s, and extension at 72 °C for 120 s. The first PCR product was subjected to the second PCR 10 cycles with Illumina TruSeq P5 and Index-containing P7 adapters. After purification using a MinElute Gel Extraction Kit (Qiagen, Inv., Valencia, CA), 16S rRNA gene sequencing was performed using the Illumina MiSeq Reagent Kit v2 by Illumina MiSeq System (Illumina, San Diego, USA) with standard Illumina software. The paired-end sequence reads were demultiplexed, trimmed, quality filtered, and chimera removed. The screened reads were processed by Qiime2 version 2022.2 [34] and DADA2 version 1.5.2 [35]. SILVA SSU Ref NR database version 138.1 [36] in the Qiime2 program was used for alignment and taxonomic affiliation of the representative amplicon sequence variants (ASVs). Rarefaction analysis was performed for the ASVs using the Vegan: Community Ecology in R version 4.4.1 (R Development, Core Team, 2013) [37, 38].

To correlate 16S rRNA gene sequences in the amplicon library and near-complete genomes reconstructed from groundwater samples from the Horonobe URL [5], a BLASTn search was performed against RefSeq prokaryote representative genomes [39]. Near-complete genomes with 16S rRNA gene sequences closely related to dominant populations in the amplicon library were subjected to protein function annotation by the Kyoto Encyclopedia of Genes and Genomes (KEGG) pathway tool [40], along with the BlastKOALA tool [41]. The taxonomic classification was performed for the near-complete genomes according to the Genome Taxonomy Database (GTDB) taxonomy [42]. For aligning protein sequences, the MAFFT online service was used [43]. The resulting alignment was used to construct the maximum likelihood phylogenetic tree using W-IQ-TREE [44] with 1000 replicates of ultrafast bootstrap [42, 44, 45]. The tree was visualized using FigTree v1.4.4 (http://tree.bio.ed.ac.uk/software/figtree/).

## Results and Discussion

### Hydrogeological Characteristics of EDZ Fractures

Around the Horonobe URL, hydrogeological characterizations have been conducted using drilled rock cores and boreholes with multi-packer systems [25, 26, 29, 46, 47]. During the construction of the 350-m galleries, geological features and groundwater inflow were examined for excavated gallery walls [48]. The drilling point at the 350-m gallery was located near a zone fractured by faulting [49]. In this study, whole-round rock cores were obtained by drilling a 2-m-long horizontal borehole from a gallery wall, where a hydraulic test was performed by injecting water through a single-packer system. BTV logging revealed the fracture density was high at a 17 − 55-cm section (fractures named BTV1-8; Supplementary Fig. 1 and Table S1; [50]). The ranges of strikes and dips at the 17 − 55-cm section were consistent with the formation of fractures in EDZ (Table S1) when the direction of excavation advancement was southwest. Hydraulic conductivities were measured by the hydraulic test (Table S2). Sections 30 − 200 cm and 45 − 200 cm were associated with a high hydraulic conductivity of 1.50 × 10^−6^ m/s, whereas the other sections had lower hydraulic conductivity ranging from 7.6 × 10^−10^ to 1.9 × 10^−7^ m/s. These results indicate that the fractures in the 17 − 55-cm section are open and permeable. Core observations revealed a dark brown rock color, no bioturbation, and 27 fractures (Supplementary Fig. 1 and Table S3). The examination of a 2-m-long rock core sequence with a 0 − 15.5-cm section of cementing material revealed that the edges of fractures in the drill cores were sharp (e.g., no secondary fractures/cleavage along the fracture surface; Supplementary Fig. 1 and Table S3), which is a characteristic of fractures formed by extensional force in EDZ rather than deformed by fault-related compressional shearing [51, 52]. Thus, these characteristics all support the fact that the fractures at the 17 − 55-cm section were formed by the gallery excavation.

### Biofilm Formation at EDZ Fractures

**Fig. 2:**
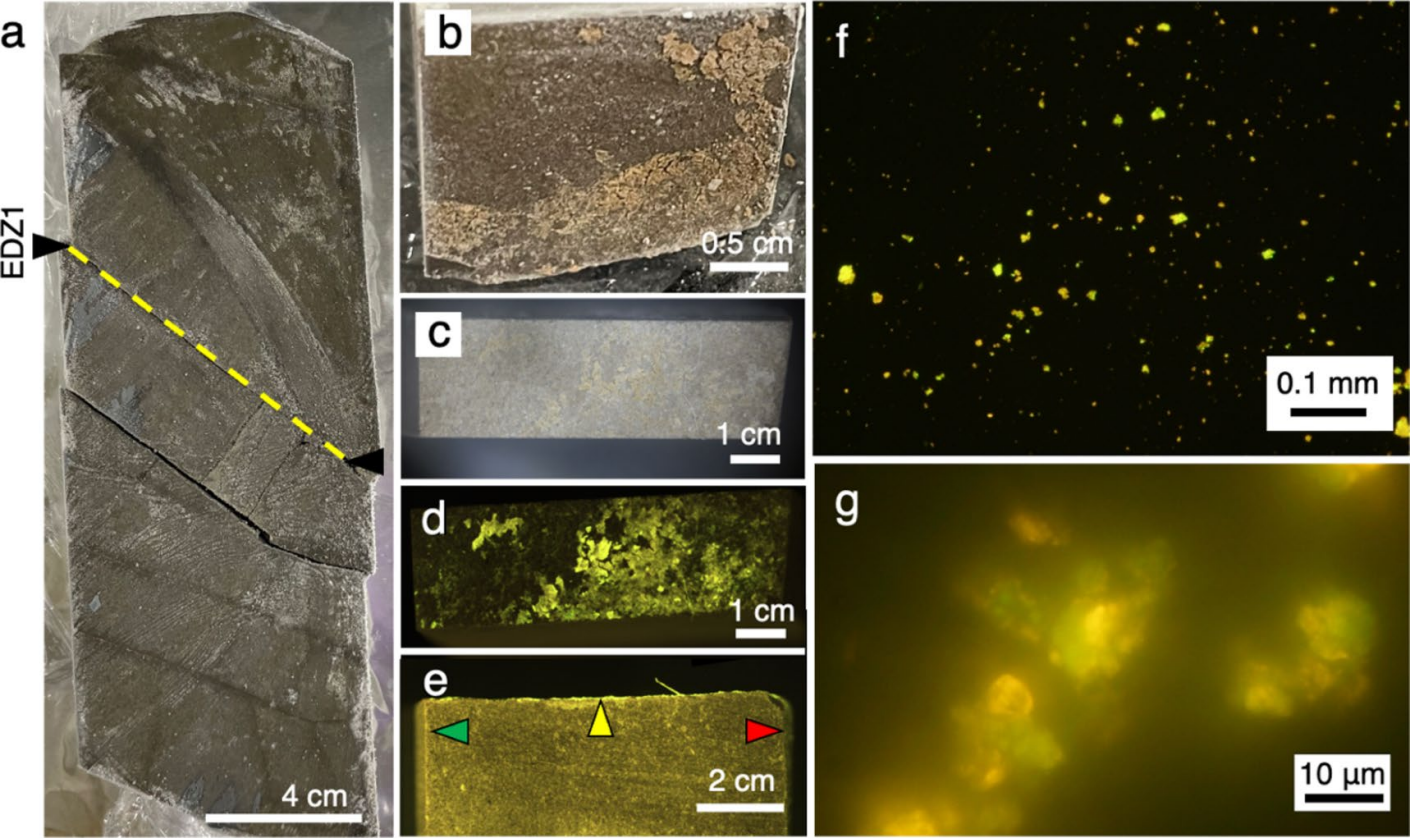
Photographs of a half-split core sequence from ~0.3 to ~0.5 mabh (**a**), the fracture surface at EDZ1 on the lower block (**b**), and the fracture surface at EDZ1 created on the lower block by cutting with a band saw (**c**). Fluorescence stereo microscopic images of the fracture surface at EDZ1 from the top (**d**) and the side (**e**). The yellow arrow and yellow dotted line indicate the fracture at EDZ1 (**a**, **e**). Green and red arrows indicate the surfaces created by cutting with a band saw and drilling, respectively (**e**). Fluorescence microscopic images of the material detached from the fracture surface (**f**, **g**)

We characterized the frozen core samples in which a light brownish film extensively covered one of the EDZ fractures at ~36 cm (BTV5 in Table S1, Fracture C7 in Table S3 and EDZ1 in Fig. 2a and 2b). Greenish signals from the film stained by SYBR-Green I was observed using a fluorescence stereo microscope (Fig. 2c and 2d). To exclude the possibility that greenish signals resulted from the introduction of microbial contamination during drilling and cutting with the diamond band saw, we observed the other sides of the core block after DNA staining (Fig. 2e). In contrast, greenish signals were not evident on the surfaces caused by drilling and cutting. This result is important for clarifying that the greenish signals were not derived from contamination. After the dispersion of the film detached from the fracture into the solution, the suspension was stained by SYBR-Green I and observed by a fluorescence microscope. The aggregation of coccoid cells was associated with the minor presence of rod-shaped cells (Fig. 2f and 2g).

**Fig. 3:**
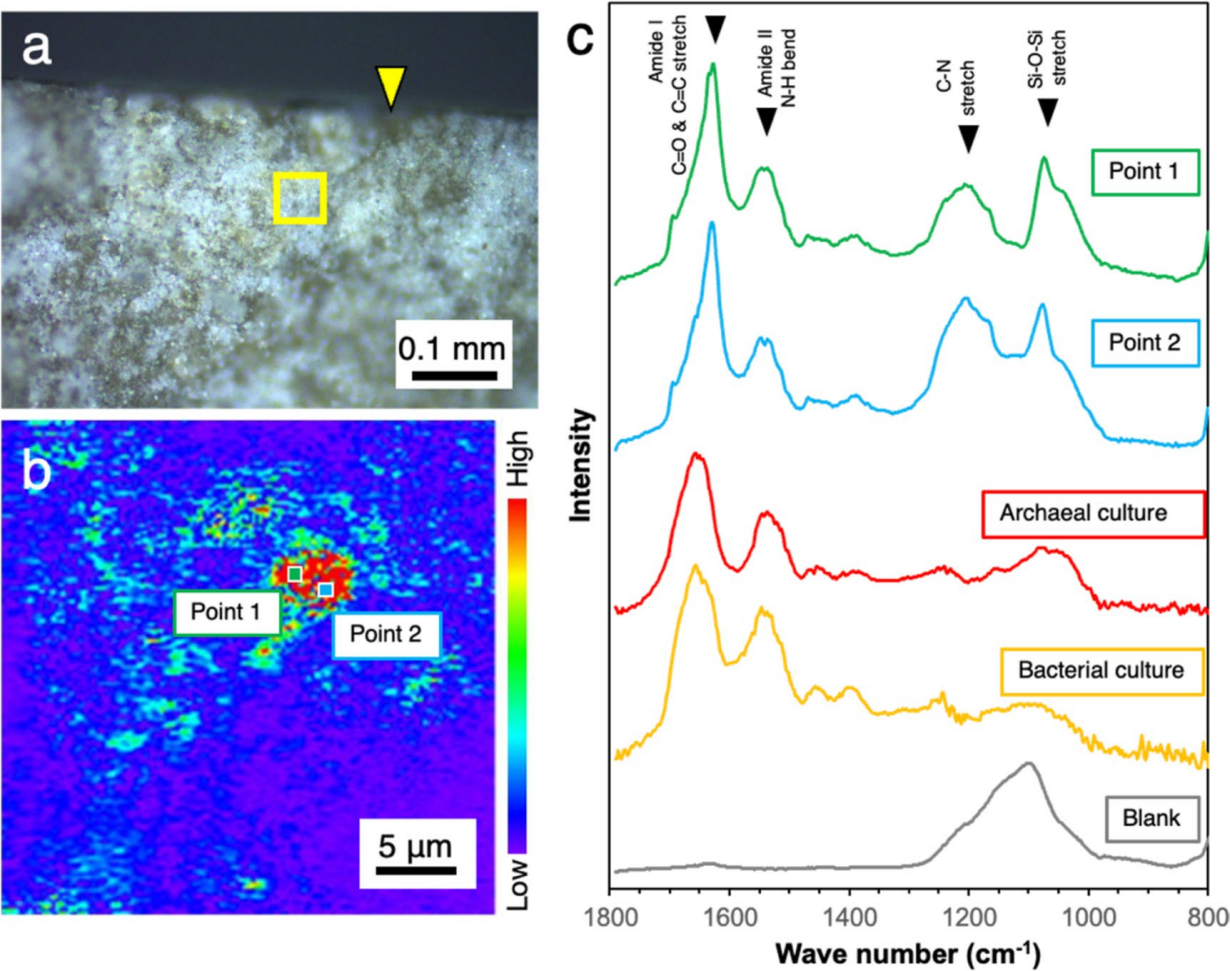
Submicron-scale spectroscopic analysis of an EDZ fracture (EDZ1). A photograph of the fracture surface (**a**), a counter map of the fracture surface highlighted with a yellow square based on the peak at 1530 cm^−1^ in optical photothermal infrared (O-PTIR) spectra (**b**). O-PTIR spectra of points 1 and 2 and cultured cells of *Nanobdella aerobiophila* strain MJ1^T^ (=JCM33616^T^) and *Metallosphaera sedula* strain MJ1HA (=JCM33617) for an archaeal reference and *Shewanella oneidensis* strain MR-1^ T^ (=ATCC 700550.^T^) for a bacterial reference (**c**). The peak assignment was based on Ellerbrock et al. and Movasaghi et al. [53, 54]. A blank spectrum was obtained from the surface created by cutting with a diamond band saw, especially where the cut surface intersected the EDZ fracture (indicated by the yellow arrow in (**a**))

To confirm whether the greenish signals originated from microbial cells rather than materials strongly bound to SYBR-Green I and/or autofluorescence, the film attached to the fracture was directly analyzed by an in situ infrared (IR) spectroscopy (Fig. 3). IR spectra characterized by two amide peaks at ~1530 and ~1640 cm^−1^ were very similar to those from microbial cultures. The amide I peak at~1640 cm^−1^ is shifted in peak position due to the difference in chemical form, which is why the peaks are slightly shifted in the four waveforms (Fig. 3).

**Fig. 4:**
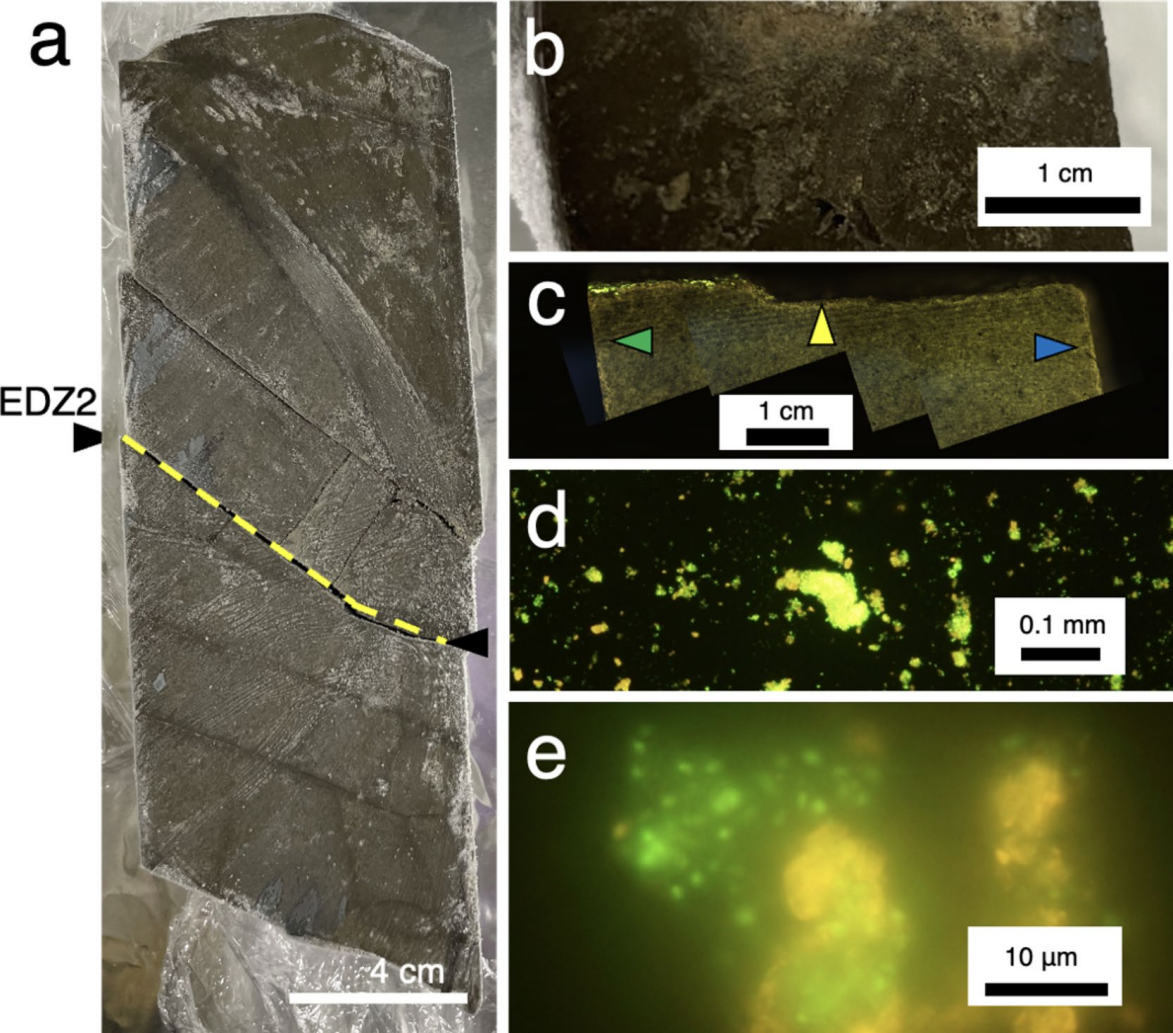
Photographs of a half-split core sequence from~0.3 to~0.5 mabh (**a**) and the fracture surface at EDZ2 on the upper block (**b**). A fluorescence stereo microscopic image of the fracture surface at EDZ2 from the side (**c**). The yellow arrow and yellow dotted line indicate the fracture at EDZ2. Green and blue arrows indicate the surfaces created by cutting with a band saw and half-splitting of the core, respectively. Fluorescence microscopic images of the material detached from the fracture surface (**d**, **e**)

**Fig. 5:**
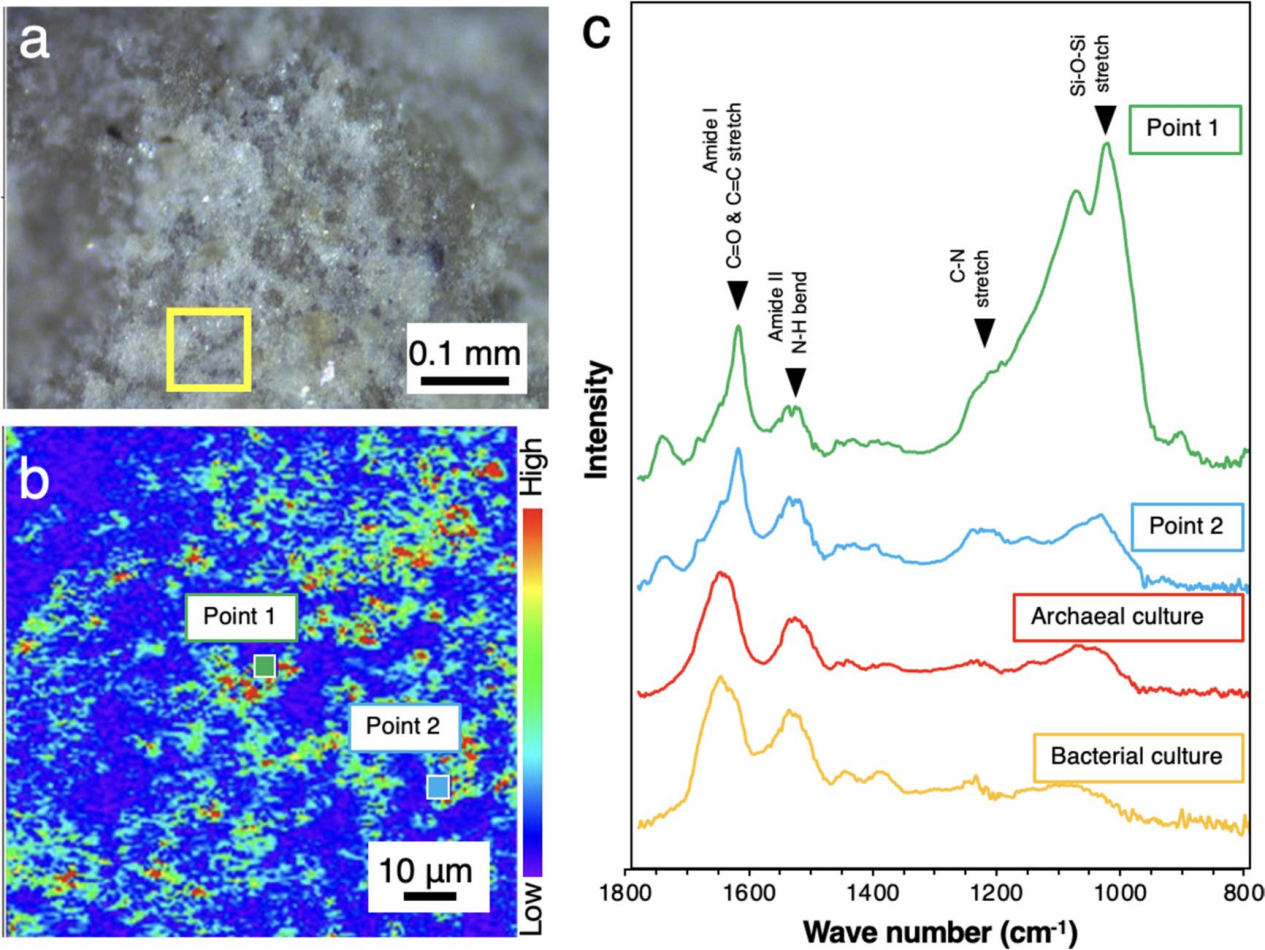
Submicron-scale spectroscopic analysis of an EDZ fracture (EDZ2). A photograph of the fracture surface (**a**), a counter map of the fracture surface highlighted with a yellow square based on the peak at 1530 cm^−1^ in optical photothermal infrared (O-PTIR) spectra (**b**). O-PTIR spectra of points 1 and 2 and cultured cells of *Nanobdella aerobiophila* strain MJ1^T^ (=JCM33616^T^) and *Metallosphaera sedula* strain MJ1HA (=JCM33617) for an archaeal reference and *Shewanella oneidensis* strain MR-1^ T^ (=ATCC 700550.^T^) for a bacterial reference (**c**). The peak assignment was based on Ellerbrock et al. and Movasaghi et al. [53, 54]

A light brownish film was also visible at an EDZ fracture at~39 cm next to EDZ1 (BTV6 in Table S1, Fracture C8 in Table S3 and EDZ2 in Fig. 4a). Greenish signals were observed at EDZ2 after SYBR-Green I staining (Fig. 4b). The absence of greenish signals at the surfaces generated by cutting with the band saw and splitting the core into half excluded the possibility of microbial contamination during core processing. Material scratched from the EDZ2 surface was also stained with SYBR-Green I. Similarly, to EDZ1, coccoid cells were aggregated (Fig. 4). The in situ IR spectroscopy also confirmed the spectra diagnostic of microbial cells at the EDZ2 (Fig. 5). These results clarified the biofilm formation at the EDZ fractures.

In addition to the EDZ fractures, fractures were visible in the half-split core sequence from approximately 0.3 to 0.5 mabh (Supplementary Fig. 2). However, these fractures were not observed in the borehole (Table S1), indicating that their formation was likely caused by drilling. The materials scraped from two of the drilling-induced fractures (Fractures C9 and C10 described in Table S3 and indicated by a yellow and a pink arrow, respectively, in Supplementary Fig. 2a) were stained with SYBR-Green I. However, microbial cells were not visualized by fluorescence microscopy (Supplementary Fig. 2b–c).

### Taxonomic Profiling of Biofilm Communities at EDZ Fractures

**Fig. 6:**
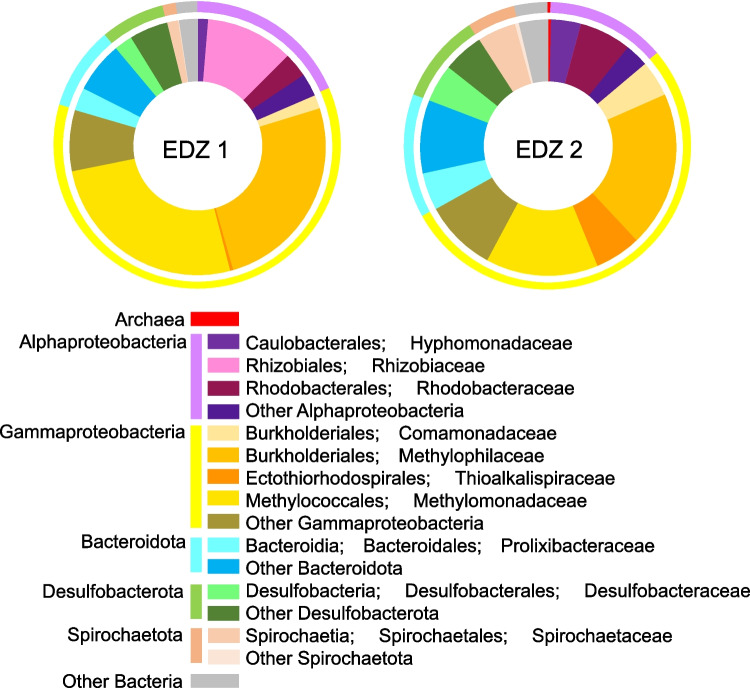
Microbial community structures from biofilms on fractures named EDZ1 and EDZ2. The phylum and class were classified by 16S rRNA gene sequences in the nomenclature based on the SILVA 138 database using QIIME2 software

16S rRNA gene amplicon analysis was performed for materials aseptically scratched from EDZ1 and EDZ2 (Fig. 6 and Table S4). We obtained 3119 and 2939 sequences from EDZ1 and EDZ2, respectively. Rarefaction curves from EDZ1 and EDZ2 were close to the saturation levels, suggesting that EDZ2 was more diverse than EDZ1 (Supplementary Fig. 3). Although ten archaeal sequences were obtained from EDZ2, the archaeal sequences were not affiliated with anaerobic methane-oxidizing archaea. In both samples, the most dominant taxonomic group was the family Methylophilaceae of Gammaproteobacteria. The proportion of the family Methylomonadaceae was similarly high in EDZ1, whereas those of the families Rhodobacteraceae and Desulfobacteraceae were relatively high in EDZ2. All cultivated members of Methylomonadaceae (the genera *Methylobacter*, *Methyloglobulus*, *Methylomarinum*, *Methylomicrobium*, *Methylomonas*, *Methyloprofundus*, *Methylosarcina*, *Methylosoma*, *Methylosphaera*, and *Methylovulum*) and Methylophilaceae (the genera *Methylobacillus*, *Methylophilus*, *Methylotenera*, and *Methylovorus*) are aerobic methanotrophs [55, 56].

We also performed the 16S rRNA gene amplicon analysis for the materials scraped from the surfaces created by drilling (the red arrow in Fig. 2e) and cutting with a diamond band saw (the green arrows in Figs. 2e and 4c). However, PCR amplification was not successful. These results exclude the possibility that 16S rRNA gene sequences obtained from the EDZ fractures did not originate from contamination from the drilling and the rock interior.

### Genomic Features of Aerobic Methanotrophs at the Horonobe URL

It is straightforward to obtain metagenomic data from the same DNA, from which 16S rRNA gene sequences were obtained to clarify metabolic capabilities of the dominant microbial populations at the EDZ fractures. However, metagenomic analysis was not performed due to the small sample volumes of the materials scraped from the EDZ fractures. Instead, we searched metagenomically assembled genomes (MAGs) previously reconstructed from groundwater samples from underground boreholes at the Horonobe URL [5]. The top ten 16S rRNA gene sequences (EDZ_1~10 in Table S4), which taxonomically cover 75% of EDZ1 (2347/3119 reads) and 55% of EDZ2 (1622/2939 reads), were compared to 16S rRNA gene sequences in the MAGs. However, none of the 16S rRNA gene sequences has closely related 16S rRNA gene sequences in the MAGs, except for those affiliated within the families Methylophilaceae and Methylomonadaceae of Gammaproteobacteria (EDZ1, 2, and 4). We found gammaproteobacterial MAGs containing 16S rRNA gene sequences nearly identical to those affiliated with the Methylomonadaceae and Methylophilaceae in the 16S rRNA gene amplicon libraries (Tables S5 and S6). Based on the Genome Taxonomy Database (GTDB) taxonomy, the classifications of the MAGs with the nearly identical 16S rRNA gene sequences were UBA6140 of the Methylophilaceae and PGZD01 of the Methylomonadaceae (Table S5).

**Fig. 7:**
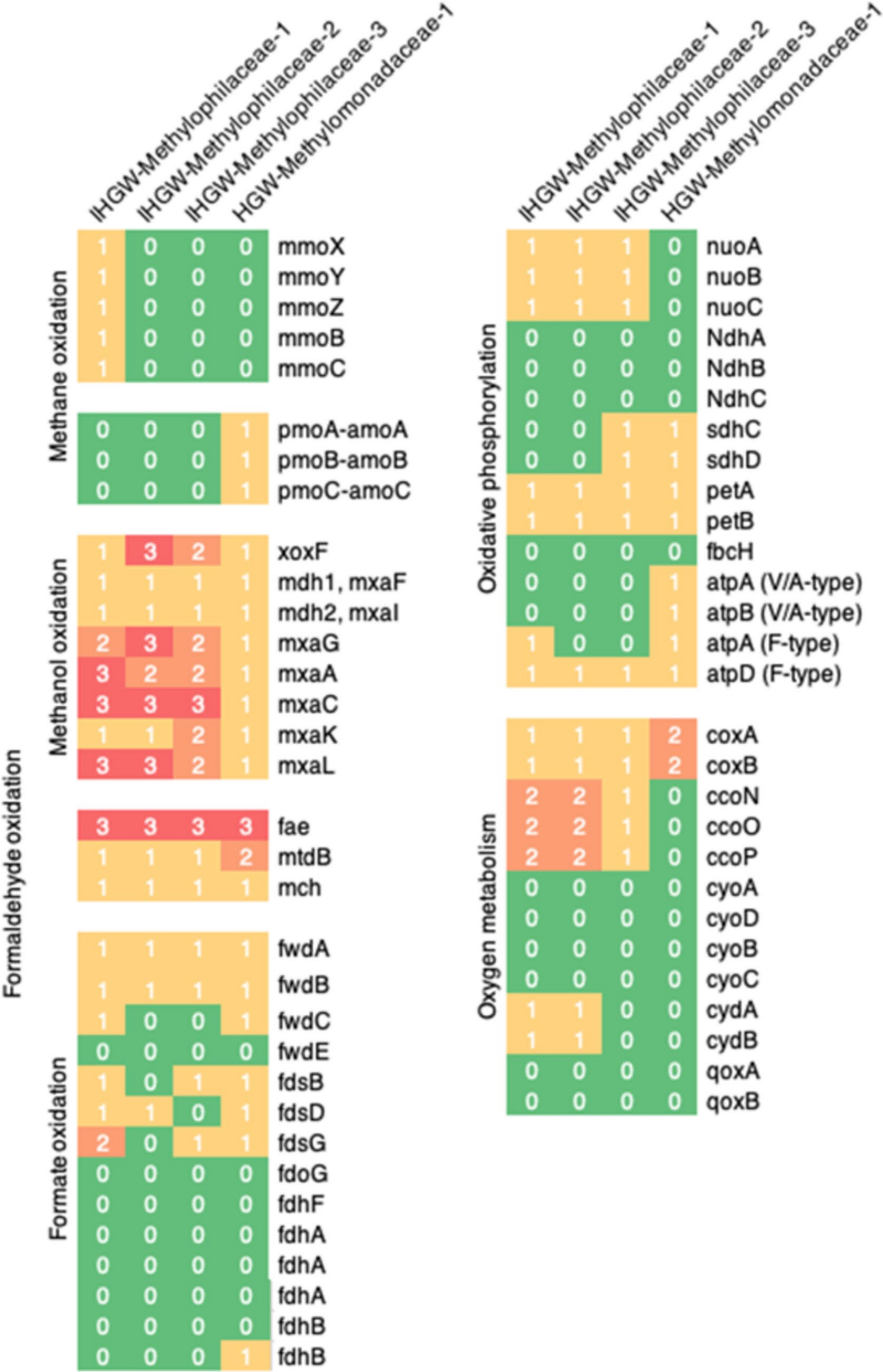
An overview of the presence or absence of metabolic genes for energy generation via aerobic methanotrophy in Methylophilaceae- and Methylomonadaceae-affiliated Horonobe groundwater MAGs. Gene annotation was performed using BlastKOALA with default thresholds (Table S7)

Three Methylophilaceae-affiliated MAGs and one Methylomonadaceae-affiliated MAG were subjected to gene function analysis by the KEGG pathway tool. For methane oxidation, key genes encoding methane oxidation are curated according to MCycDB [57] (Fig. 7 and Table S7). While one of the Methylophilaceae-affiliated MAGs had a gene cluster for sMMO, a soluble diiron monooxygenase family enzyme [58], genes for particulate methane monooxygenase (pMMO) are present in the Methylomonadaceae-affiliated MAG. Next to methane oxidation, methanol is oxidized to formaldehyde by dehydrogenase. The Methylophilaceae- and Methylomonadaceae-affiliated MAGs had multiple genes encoding xoxF and mxaGACKL. As for formaldehyde oxidation, the Methylophilaceae- and Methylomonadaceae-affiliated MAGs contained genes involved in the HMTP/H4F-linked pathway [59]. Finally, formate is oxidized to CO_2_ potentially by enzymes encoded as fwdABC and fdsBDG. In addition, a set of genes involved in oxidative phosphorylation with O_2_ as a terminal electron acceptor is encoded in the Methylophilaceae- and Methylomonadaceae-affiliated MAGs (Fig. 7 and Table S7). Thus, aerobic methanotrophy by the members of Methylophilaceae and Methylomonadaceae at EDZ1 and EDZ2 is supported by the contents of closely related MAGs obtained from the underground groundwater samples at the Horonobe URL [5].

### Abiotic and/or Microbiologically Mediated Processes Forming Oxygenated Conditions

Recently, the abundance of aerobic methanotrophs has been demonstrated in highly reducing aquifers over a large geographic area (> 210,000 km^2^) [23]. In highly reducing groundwater samples from underground boreholes at Horonobe URL where the Methylophilaceae- and Methylomonadaceae-affiliated MAGs were reconstructed, the abundance of the Methylophilaceae- and Methylomonadaceae-affiliated MAGs were 10% and 1%, respectively, based on the coverage of rpsl3 genes [5]. Regarding the Methylophilaceae- and Methylomonadaceae-affiliated MAGs, aerobic methanotrophy is supported by near-complete genomes equipped with a full set of methane oxidation and oxidative phosphorylation with O_2_ as a terminal electron acceptor. In addition, it is established that molecular oxygen is essential to activate the catalytic site in pMMO for CH_4_ oxidation [60].

The drainage of groundwater from the rock interior to the excavated tunnel is generally associated with air intrusion before cementing the gallery wall [9]. However, no significant oxidation of the rock interior was observed around the 350-m-deep gallery of Horonobe URL after excavation [29]. This is because air intrusion into the EDZ fractures was prevented by the filling of fractures with CH_4_, which was degassed from groundwater due to the drainage-related pressure drop [29]. Another possibility is the intrusion of oxygenated solution from pre-grouting used during gallery excavation. This possibility is also unlikely because the amount of the intruded O_2_ is that dissolved in the grout-bearing water by air saturation (~ 0.3 mM). This amount of O_2_ is too small to sustain aerobic methanotrophs for 7 and a half years after the gallery excavation. Instead of introducing O_2_ from the surroundings, in situ formation of O_2_ in the EDZ fractures might be plausible when fracturing of silicate rocks produces radicals including Si^•^, SiO^•^, and SiOO^•^ on the fracture surfaces [61]. However, the possibility that atmospheric O_2_ might get into the rock through fractures and water movement, as previously demonstrated by stable isotope analysis, needs to be excluded [18].

**Fig. 8:**
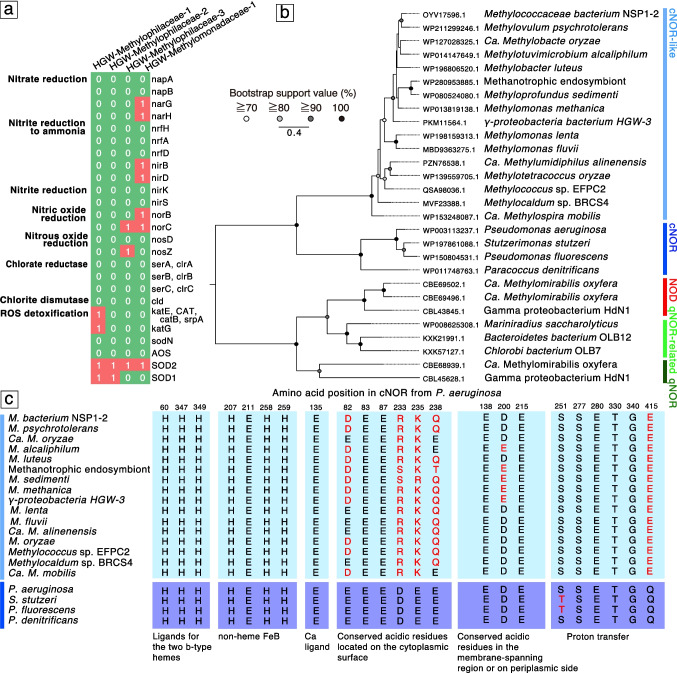
An overview of the presence or absence of metabolic genes involved in the potential production of O_2_ in Methylophilaceae- and Methylomonadaceae-affiliated Horonobe groundwater MAGs (**a**). Gene annotation was performed using BlastKOALA with default thresholds (Table S7). A maximum likelihood phylogenetic tree of nitric oxide reductases (NOR) including quinol-dependent NOR (qNOR) and NOR with a cytochrome c subunit (cNOR), qNOR-relate, cNOR-like and potential genes for NO dismutase (**b**). Amino acid sequence comparisons of cNOR motifs (**c**)

Alongside the formation of H_2_ by the reactions of the radicals with H_2_O, reactive oxygen species (ROS) such as H_2_O_2_, O_2_^−^, and OH^•^ could also be produced [62]. It is well known that aerobic organisms use enzymatic reactions (e.g., catalase or superoxide dismutase) to detoxify ROS, which often results in the generation of oxygen [63, 64]. The presence of genes for catalase or superoxide dismutase in the near-complete genomes supports the in situ production of O_2_ by the combination of the abiotic and/or microbiologically mediated processes (Fig. 8a and Table S7). Although it is likely that oxygen utilized by aerobic methanotrophs is generated in situ by the fracturing of silicate rocks, the duration and sustainability of this oxygen-producing process remain to be clarified.

In addition to the detoxification of ROS, two pathways are known for dark, non-photosynthetic oxygen production: chlorite dismutation during perchlorate and chlorate respiration (ClO_2_^−^ → Cl^−^  + O_2_) and nitric oxide (NO) dismutation occurs following its production through nitrite reduction (2NO → N_2_ + O_2_) [65]. In the large graphic aquifers, the aerobic metabolism is supported by microbial production of dark O_2_ by the dismutation of chlorite and nitric oxide [23]. Respiration with (per)chlorate requires two enzymes. At first, (per)chlorate reductase catalyzes the reduction of perchlorate to chlorate and of chlorate to chlorite. Then, chlorite is converted to chloride and oxygen (ClO_2_^−^ → Cl^−^  + O_2_) by chlorite dismutase [66, 67]. However, neither of the genes for (per)chlorate respiration was present. Although neither of the genes for (per)chlorate respiration was present in the Methylophilaceae- and Methylomonadaceae-affiliated MAGs (Fig. 8a and Table S7), other community members might be responsible for the dark O_2_ production via chlorite dismutation.

Dark O_2_ production with NO dismutation was first demonstrated by the methane-oxidizing bacterium *Candidatus Methylomirabilis oxyfera* [68]. Despite the apparent lack of an identifiable nitrous oxide reductase in the genome of *M. oxyfera*, dinitrogen gas was the end product of nitrite reduction, which suggests the possibility of a novel enzyme (NO dismutase) that catalyzes the disproportionation of NO into N_2_ and O_2_. *Ca. M. oxyfera* has three types of nitric oxide reductases (NORs) typically catalyzing the two-electron reduction of two molecules of NO into N_2_O (2NO + 2H^+^ + 2e^−^ → N_2_O + H_2_O) [65]. One of near-complete genomes of aerobic methanotrophs (HGW-Methylomonadaceae-1) was found to encode pMMO genes and a NOR gene (Fig. 8a and Table S7). Although NORs of *Ca. M. oxyfera* are all classified as quinol-dependent NO reductases (qNOR), whereas the NOR gene in HGW-Methylomonadaceae-1 was classified as NOR with a cytochrome c subunit (cNOR) as the electron donor for the catalytic reaction. The cNOR sequence from HGW-Methylomonadaceae-1 was distinct from those well characterized for the catalysis of a reaction from NO into N_2_O by *Pseudomonas aeruginosa* [69]. Instead, the cNOR sequence from HGW-Methylomonadaceae-1 was related to those from the family Methanococcaceae, all cultivated members of which are aerobic methanotrophs [69] (Fig. 8b). The structure of cNOR reveals that non-heme iron FeB and heme b_3_ constitute the binuclear center serving as the catalytic site (Fig. 8c). Amino acid residues coordinating the non-heme FeB and the heme b_3_ in cNOR from the family Methanococcaceae were identical to those in functionally characterized cNOR from *P. aeruginosa.* However, conserved amino acid residues were particularly different on the cytoplasmic surface, suggesting the deviation of catalytic activity for a reaction from NO into N_2_O. Like the putative NO dismutase proposed in *Ca. M. oxyfera*, cNOR in the family Methanococcaceae including HGW-Methylomonadaceae-1 might catalyze a reaction for the production of O_2_ and N_2_ for aerobic methanotrophy.

### Implications for Radioactive Waste Disposal

To dispose of radioactive waste in the deep subsurface, subsurface tunnels need to be excavated, which introduces O_2_ from the air into the deep geological environment. As the anoxic environment is favorable for the disposal of radioactive waste [22], microbial activity is expected to promote oxygen consumption after closing the subsurface tunnels by backfilling. In addition, microbial activity is associated with the corrosion of metal canisters and gas production by the decomposition of organic matter. In many countries, such microbial influences are therefore described in the Safety Case Report published by implementers (e.g., [20, 70–73]). Our results suggest that the oxic environment is established by microbial production of dark O_2_ within the EDZ fractures. If dark O_2_ is present as dissolved species in groundwater, redox-sensitive radionuclides (e.g., U and Np) are oxidized and potentially mobilized. Consequently, it may be necessary for implementers to conduct a safety assessment on the production of dark O_2_ by subsurface microbes.

## Conclusion

Drilling, borehole logging, and core characterizations were conducted on the EDZ fractures formed by the construction of the Horonobe URL. The biofilms were found to be mainly composed of gammaproteobacterial lineages, whose cultivated members consist exclusively of aerobic methanotrophs. Aerobic methanotrophy supported by the presence of gene sets in near-complete genomes from these lineages suggests an energetically favorable subsurface habitat within the EDZ fractures. Our future research will be directed to clarify the processes involved in the formation of oxygenated conditions and spatiotemporal changes in microbial communities and physicochemical properties with respect to mass transport through the permeable fracture network.

## Supplementary Information

Below is the link to the electronic supplementary material.

Supplementary file1 (DOCX 2.80 MB)

Supplementary file2 (XLSX 157 KB)

## Data Availability

The datasets analyzed during the current study are available from the corresponding, first and second authors upon reasonable request. Sequence data is provided within Table S4. The data will be deposited to, and then available from DDBJ upon the acceptance.
