# Supplementary material for: Biofilm Formation on Excavation Damaged Zone Fractures in Deep Neogene Sedimentary Rock
Source: Microb Ecol. 2024 Oct 22;87:132. doi: 10.1007/s00248-024-02451-7 (PMC11496357; doi:10.1007/s00248-024-02451-7)
Supplement: Supplementary file 1 [file 248_2024_2451_MOESM1_ESM.docx]

**Supplementary Information**

**Biofilm Formation on Excavation Damaged Zone Fractures in Deep Neogene Sedimentary Rock**

Akinari Hirota^1†^, Mariko Kouduka^2†^, Akari Fukuda^2^, Kazuya Miyakawa^3^, Keisuke Sakuma^4^, Yusuke Ozaki^3^, Eiichi Ishii^4^, Yohey Suzuki^2^*

^1^Regulatory Standard and Research Department, Secretariat of Nuclear Regulation Authority (S/NRA/R), 1-9-9, Roppongi, Minato-ku, Tokyo 106-8450, Japan

^2^Department of Earth and Planetary Science, The University of Tokyo, 7-3-1 Hongo, Bunkyo-ku, Tokyo, Japan

^3^Horonobe Underground Research Center, Japan Atomic Energy Agency, 432-2 Hokushin, Horonobe-cho, Hokkaido 098-3224, Japan

^4^Nuclear Safety Research Center, Japan Atomic Energy Agency, 2-4 Shirakata, Tokai-mura, Naka-gun, Ibaraki 319-1195, Japan

^†^These authors contributed equally: Akinari Hirota and Mariko Kouduka

*Corresponding author. E-mail: yohey-suzuki@eps.s.u-tokyo.ac.jp (Y.S)

Supplementary Fig. 1. Borehole and core characteristics examined on site. Borehole televiewer (BTV) images of the borehole with fractures indicated by red lines (a). The fracture features are described in Table S1. A photo image of drill cores (b). The fracture features are described in Table S3.

Supplementary Fig. 2. A photograph of a half-split core sequence from approximately 0.3 to ~0.5 mabh (a). Fluorescence microscopic images of the materials detached from the surfaces of Fracture C9 (b) and Fracture C10 (c).

Supplementary Fig. 3. Relative richness of amplicon sequence variants (ASVs) from EDZ1 and EDZ2 shown through rarefaction analysis.
